# Supplementary material for: Trait-based signatures associated with persistence and thermal benefit in a genomically decayed coral probiotic
Source: ISME J. 2026 May 7;20(1):wrag106. doi: 10.1093/ismejo/wrag106 (PMC13313319; doi:10.1093/ismejo/wrag106)
Supplement: Supplementary_material_wrag106 [file supplementary_material_wrag106.zip › Supplementary figures.pdf]

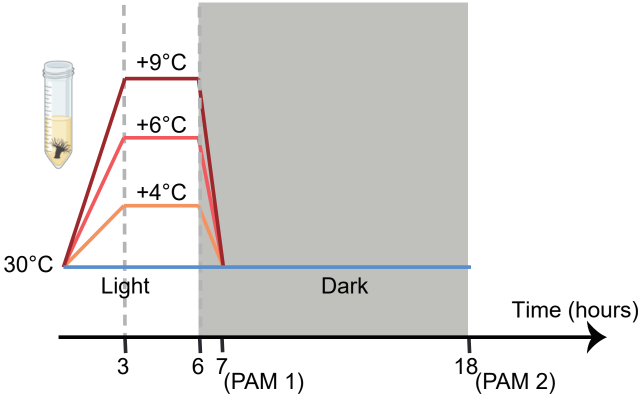

Fig. S1. Temperature profiling of the CBASS.

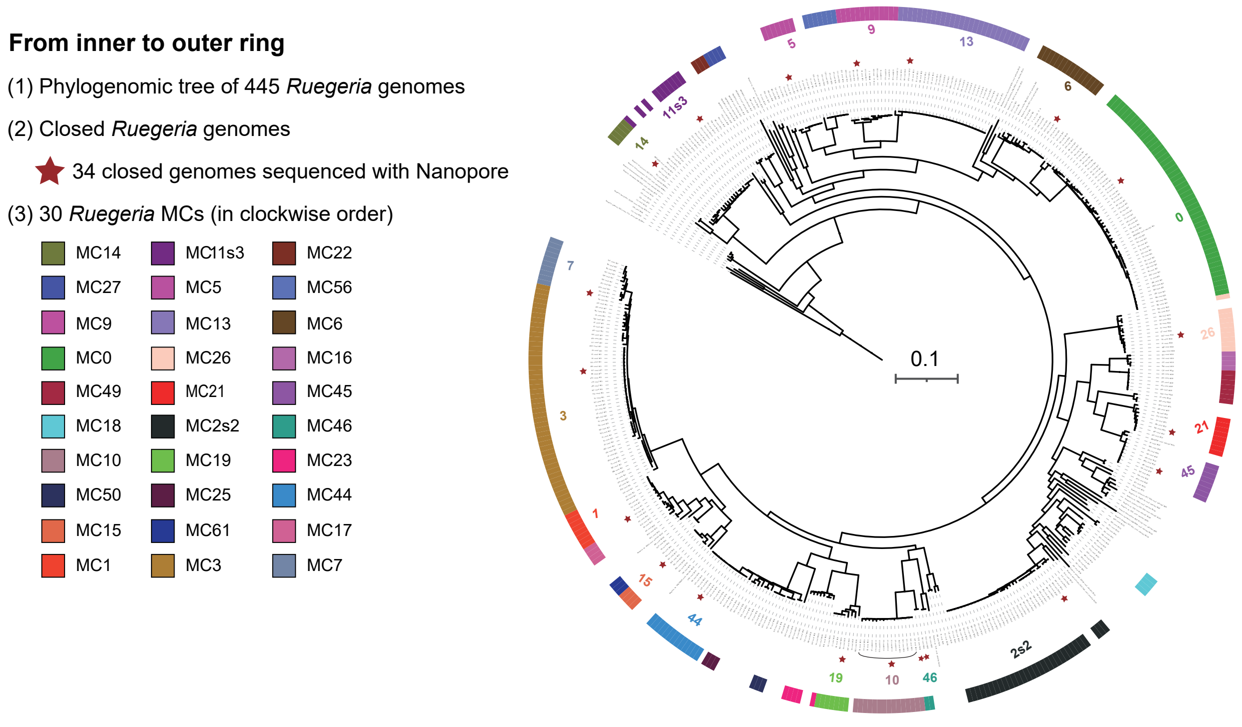

Fig. S2. **Rooted maximum-likelihood phylogenomic tree of 445 *Ruegeria* strains.** From inner to outer ring: (1) The phylogenomic tree comprising 419 newly isolated coral-associated *Ruegeria* strains and 26 publicly available genomes (NGS genomes). This tree was constructed with IQ-TREE based on concatenated single-copy orthologous genes at the amino acid level. (2) 34 closed genomes sequenced with Nanopore were labelled with stars in the tree. (3) PopCOGenT was used to delineate the *Ruegeria* populations (i.e., main clusters or MCs). 29 *Ruegeria* populations which consists of at least three isolates as defined by PopCOGenT, along with MC46, the sister group of MC10, comprising two isolates. Scale bar: 0.1.

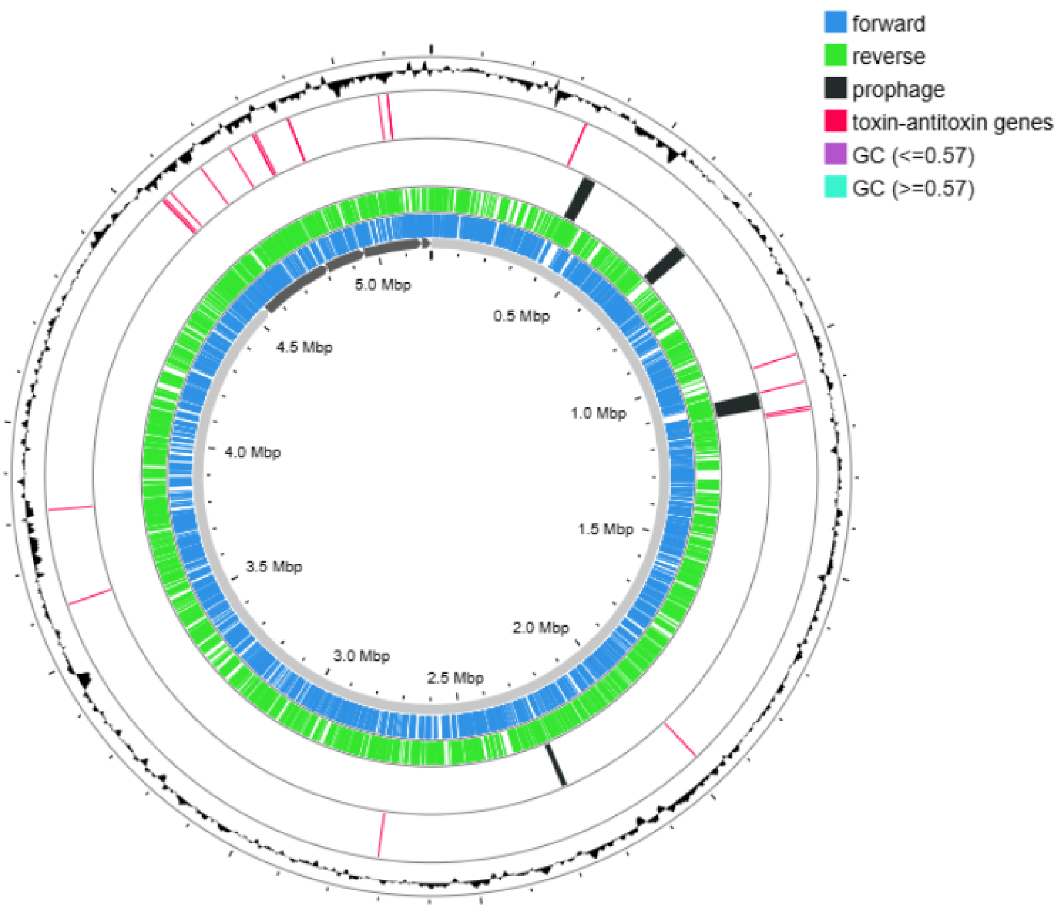

Fig. S3. Genome maps of *Ruegeria* MC10-B4. From inner to outer ring: (1) chromosomes and plasmids; (2) annotation of predicted genes on the forward strand; (3) annotation of predicted genes on the reverse strand; (4) putative prophages, (5) toxin-antitoxin genes; (6) GC skew (outer: GC ≥ 0.57; inner: GC < 0.57).

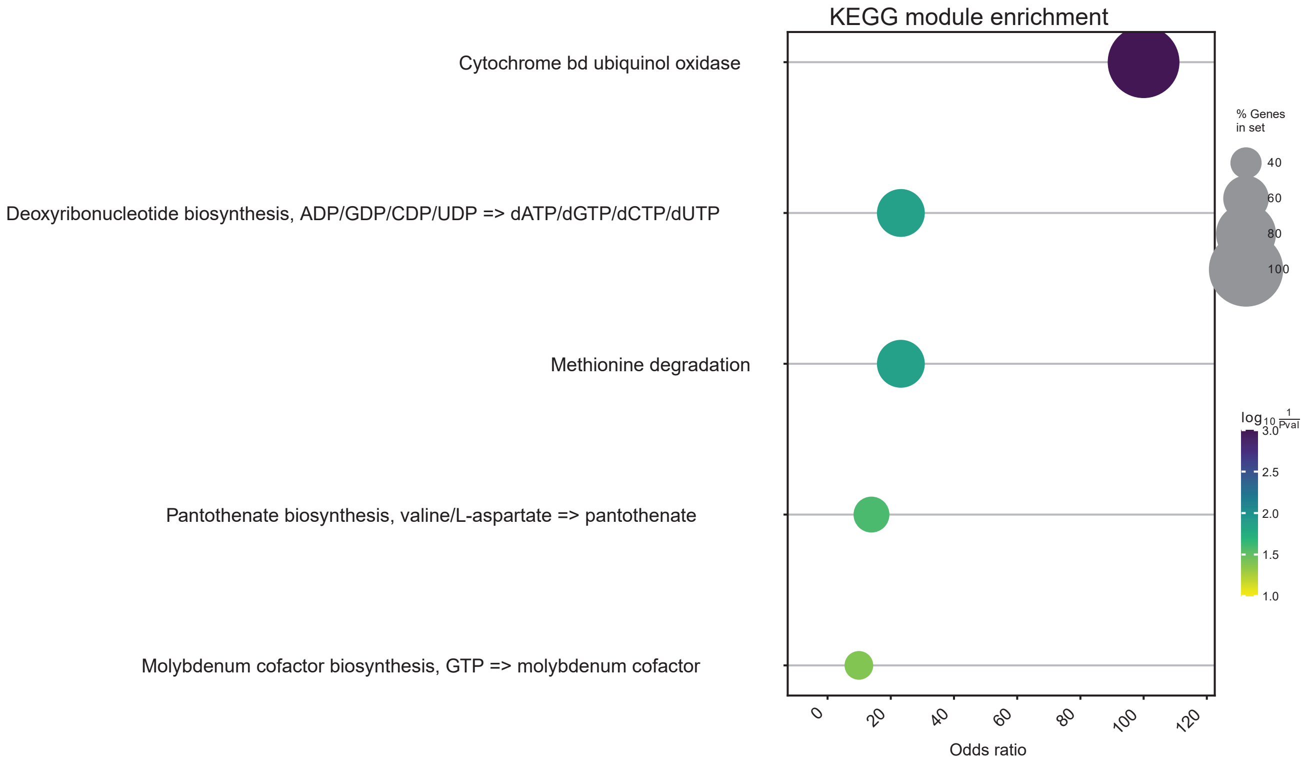

Fig S4. KEGG module enrichment analysis of MC10-specific genes. The bubble plot illustrates functional modules significantly enriched in the MC10 members (n=15) compared to the other *Ruegeria* populations (n=20). Y-axis shows KEGG Module Descriptions; X-axis represents the Odds Ratio, indicating the strength of functional enrichment. The dot size is proportional to the number of gene hits, reflecting functional dosage. The color indicates the Adjusted P-value derived from GSEApy (hypergeometric test). All displayed moduless satisfy the dual criteria of GSEApy P-value < 0.05 and PGLS P-value < 0.05.

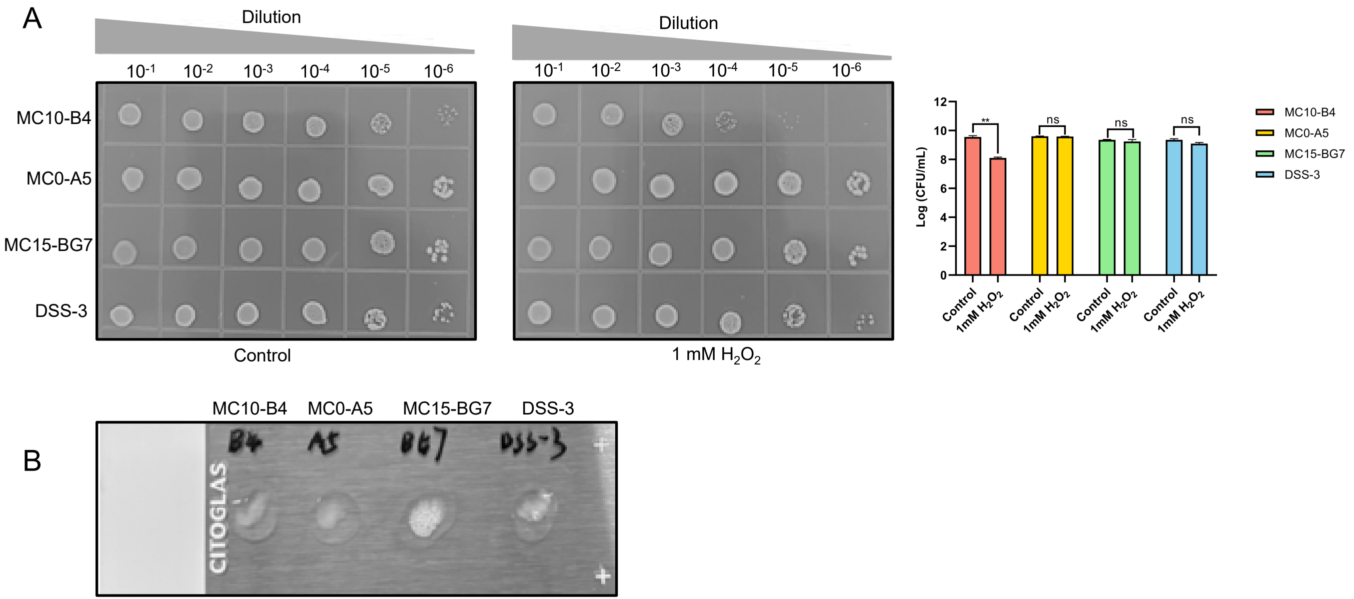

Fig. S5 H_2O_2 sensitivity and catalase activity in *Ruegeria* strains. (**A**) The ability of the indicated strains to survive in the presence and absence of 1 mM H_2O_2. Exponential-phase cultures were harvested, adjusted to an OD_{600} of 0.5, and then incubated for 1 hour at 28 °C with or without (control) 1 mM H_2O_2. After incubation, cells were serially diluted, spread on marine broth 2216 agar , and incubated at 28 °C for 3 days. The representative plate images are shown. The right panel quantifies the bacterial counts (Colony Forming Units, CFU) from three independent experiments. Data presented as mean ± SD. **, $P < 0.01$; ns, no significant. (**B**) Catalase activity was directly visualized using a slide catalase test. Bacterial colonies were transferred to a glass slide and mixed with 10 μL 3% H_2O_2. The immediate formation of oxygen bubbles was observed and photographed, indicating the presence of catalase enzyme.
